# First person – Alessandro A. Bailetti

**DOI:** 10.1242/dmm.040535

**Published:** 2019-05-30

## Abstract

First Person is a series of interviews with the first authors of a selection of papers published in Disease Models & Mechanisms (DMM), helping early-career researchers promote themselves alongside their papers. Alessandro Bailetti is first author on ‘Enhancer of polycomb/Tip60 represses hematological tumor initiation by negatively regulating JAK/STAT pathway activity’, published in DMM. Alessandro conducted the research described in this article while a graduate assistant in Dr Erika A. Bach's lab at New York University (NYU) School of Medicine, New York, USA. He is now a postdoctoral fellow in the lab of Dr Anthony Oro at Program in Epithelial Biology, Department of Dermatology, Stanford University, Stanford, USA, investigating development, genetics, genomics, cell signaling, gene transcription and chromatin modification.


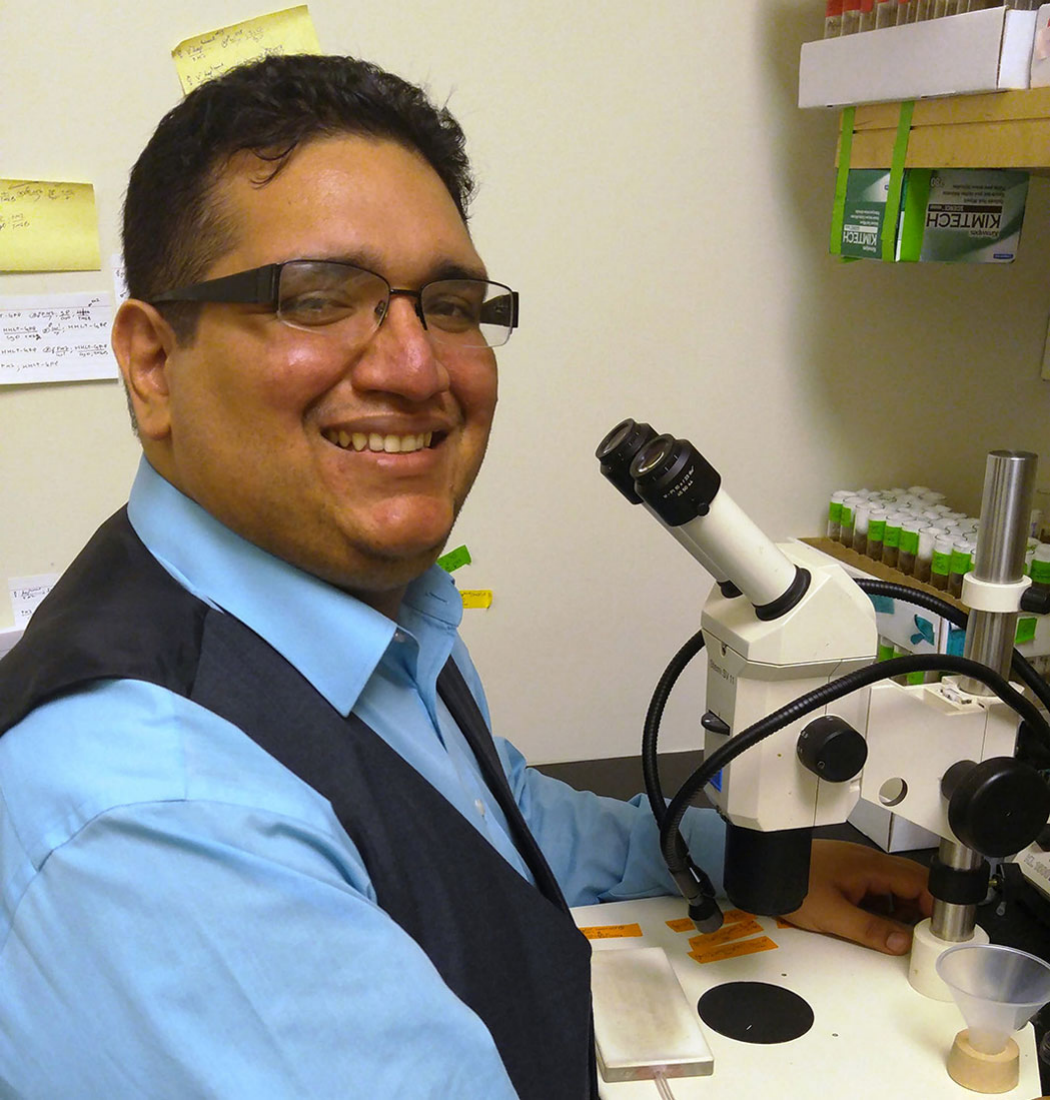


**Alessandro A. Bailetti**

**How would you explain the main findings of your paper to non-scientific family and friends?**

Myeloproliferative neoplasms are blood stem cell diseases characterized by an increased number of blood cells. Most patients have a driving mutation at their *JAK2* gene locus. A similar mutation is found in the *Drosophila* *JAK2* homolog called *hopscotch* (*hop*). Our *Drosophila* leukemic model is based on the *hop^Tumorous-lethal^* (*hop^Tum^*) mutation, which mimics the human *JAK2* mutation. These mutants have an increased number of blood cells compared to non-mutants and develop black masses, called melanotic tumors. These abnormalities are due to an increase in JAK/STAT signaling, a paramount cell-cell signaling pathway in humans, *Drosophila* and many other animals. Our findings show that *Enhancer of polycomb* [*E(Pc)*], through the Tip60 complex, inhibits blood tumor formation by inhibiting the JAK/STAT pathway and regulating the available levels of the Hop protein.

**What are the potential implications of these results for your field of research?**

Using a *Drosophila* leukemic model, our results indicate a possible connection between *E(Pc)* and the Tip60 complex, and leukemia (a previously undiscovered link). In the past few years, several other labs have used *Drosophila* to model diseases and find new therapies for them. Our findings match the observations from other *in vitro* studies. This implies the potential to use our *hop^Tum^* model to find new treatments for human myeloproliferative neoplasms.

**What are the main advantages and drawbacks of the model system you have used as it relates to the disease you are investigating?**

Working with *Drosophila* offers many advantages, such as their short life span, great number of genetic tools and evolutionary conservation. The *Drosophila* life cycle takes 10 days from embryogenesis to adulthood. To study *Drosophila*, publicly available mutant strains, tissue-specific drivers and transgenic lines to overexpress or deplete genes of interests are available. Pathways, complexes and processes in *Drosophila* are evolutionarily conserved. In our case, the JAK/STAT signaling pathway is completely conserved, but simplified. While in mammals we have multiple ligand receptors, JAKs and STATs, *Drosophila* only has a few ligands, one receptor, one JAK and one STAT. An additional advantage of working with *Drosophila* is the genetic and model organism community. We have established a well-organized, crossed-organismal community that aids the advancement of science.

“An additional advantage of working with *Drosophila* is the genetic and model organism community.”

The biggest drawback for my study was the lack of research performed in my gene of interest, *E(Pc)*. Across organisms, including humans, little is known about the role of *E(Pc)* in cells. Very few studies have focused on the biochemical characteristics of E(Pc) and its relationship to diseases and cancer. To counter this problem, I used the genetics community. In 2016, I was able to attend The Genetics Allied Conference. Since there were not many labs studying *E(Pc)* in *Drosophila*, I took advantage of the diversity of model organisms in this conference and was able to connect with a fellow graduate student who was working with the *E(Pc)* homolog in yeast. Thanks to this connection, I was able to have a colleague to discuss the current literature and increase my knowledge about E(Pc) and Tip60.

**Figure:**
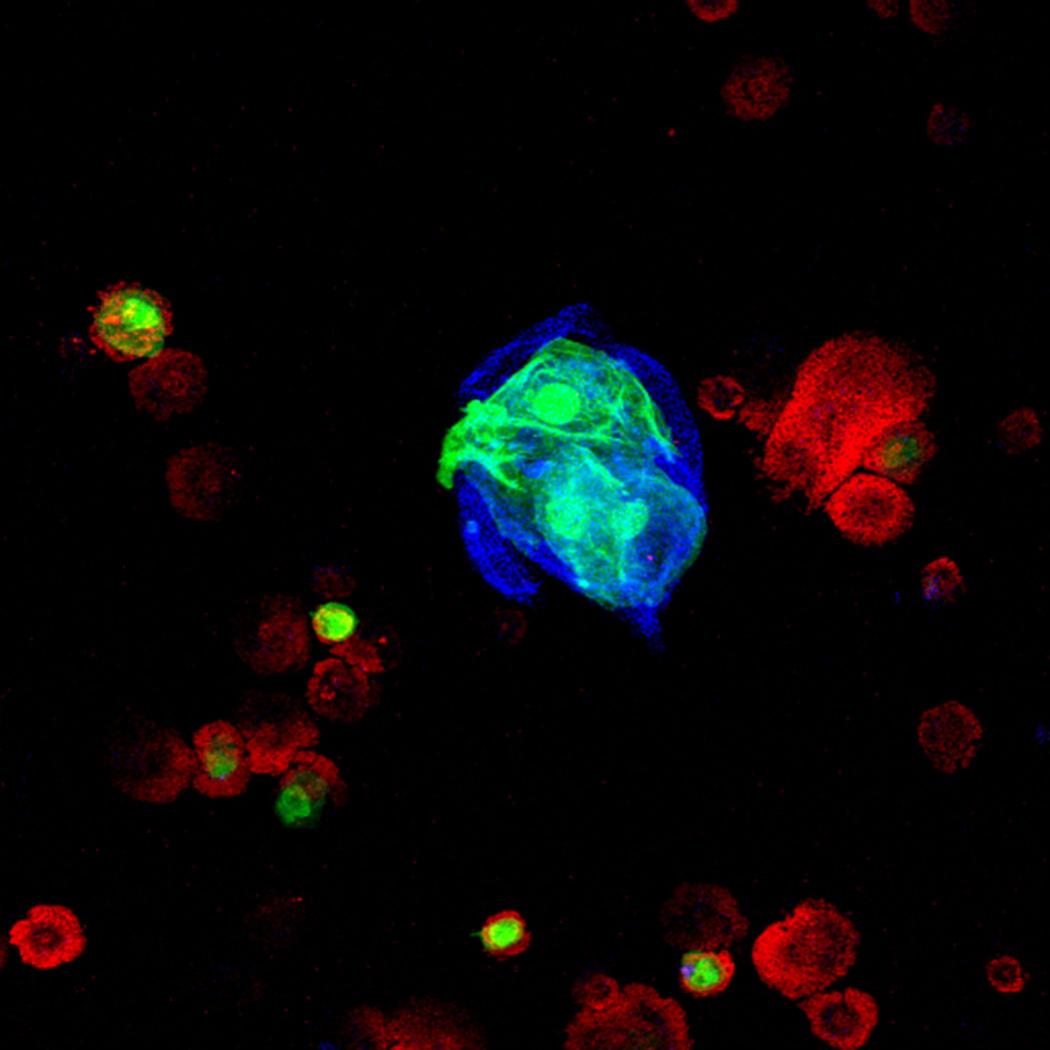
**Blood smear from *E(Pc)* knockdown animal in a wild-type background.** Depletion of *E(Pc)* is enough for lamellocyte (flatter, larger cells) differentiation, which is absent in healthy wild-type animals. In *E(Pc)*-depleted animals, hemocytes (blood cells) start aggregating. Green, *HHLT-GFP* hematopoietic driver; red, plasmatocytes; blue, F-actin.

**What has surprised you the most while conducting your research?**

As mentioned before, *hop^Tum^* mutants present blood tumors (called melanotic tumors). My biggest surprise during my research was that depletion of a single scaffolding protein such as *E(Pc)* can phenocopy the *hop^Tum^* mutations. E(Pc) is a scaffold member of the Tip60 lysine acetyltransferase complex. Surprisingly, while depleting other members of the Tip60 complex gives rise to tumor formation, depletion of E(Pc) is the most penetrant of all. Furthermore, next to depletion of *E(Pc)*, depletion of *Tip60*, the catalytic subunit of the complex, has a similar penetrance.

**Describe what you think is the most significant challenge impacting your research at this time and how will this be addressed over the next 10 years?**

In the *Drosophila* hematopoiesis field, the community has not been able to successfully perform and publish a complete transcriptome analysis of hemocytes (blood cells). In fact, 2017 saw the first study where researchers successfully performed RNA-seq on embryonic hemocytes (Bae et al., 2017). Owing to the small number of hemocytes during larval development and even during adulthood, isolating hemocytes and performing RNA-seq has been challenging. However, I see the field overcoming this challenge soon. With new single-cell RNA-seq (scRNA-seq) and cell sorting technologies, I am sure we will soon have a complete transcriptome of hemocytes. Having an available hemocyte transcriptome would aid in the understanding of transcriptional changes due to disease or infection.

“With new single-cell RNA-seq (scRNA-seq) and cell sorting technologies, I am sure we will soon have a complete transcriptome of hemocytes.”

**What changes do you think could improve the professional lives of early-career scientists?**

While being a graduate student, I have seen many changes implemented by the scientific community to improve the career development of early-career scientists. Many of my fellow early-career scientists have taken advantage of these new opportunities to find their professional niche. In my opinion, one of the biggest concerns many minority early-career scientists have is that many of them never had a minority professor as a mentor. In other words, they do not see their identity reflected in the professors they had in college or grad school. Thus, many of my fellow minority early-career scientists turn away from academics. Many of them decided to move away due to the bureaucratic system, and the unwelcoming environment. Pushing away these valuable scientists from academic careers is a disservice to the scientific community, which only continues to fuel the problem. We need minority students to follow into academic careers to become the mentors we need for future minority and diverse scientists.

“We need minority students to follow into academic careers to become the mentors we need for future minority and diverse scientists.”

**What's next for you?**

I am currently a postdoctoral fellow at Stanford University working in the lab of Dr Oro. This lab focuses on understanding the genomic organization of pluripotent stem cells during skin development and disease progression. I am excited to learn new techniques and skills I haven't used before, such as CRISPR-Cas9 genome editing and ATAC-seq, among others. In the long term, I hope to take everything I have learned at NYU and Stanford to pursue exciting new research topics in developmental and stem cell biology. I would like to open my own academic research lab. While pursuing my research, I would like to mentor and teach the next generation of scientists and hope to be a role model for fellow minority, ‘non-traditional’ and immigrant students.
